# Digital Innovations for Occupational Safety: Empowering Workers in Hazardous Environments

**DOI:** 10.1177/21650799231215811

**Published:** 2024-01-09

**Authors:** Joana Eva Dodoo, Hosam Al-Samarraie, Ahmed Ibrahim Alzahrani, Maria Lonsdale, Nasser Alalwan

**Affiliations:** 1 Department of Business Studies, College of Distance Education, Cape Coast University, Cape Coast, Ghana; 2 School of Design, University of Leeds, Leeds, UK; 3 Centre for Instructional Technology and Multimedia, Universiti Sains Malaysia, Penang, Malaysia; 4 Computer Science Department, Community College, King Saud University, Riyadh, Saudi Arabia

**Keywords:** technology and safety, hazardous occupations, workers health, immersive technology

## Abstract

**Background:**

The quest to increase safety awareness, make job sites safer, and promote decent work for all has led to the utilization of digital technologies in hazardous occupations. This study investigated the use of digital innovations for safety and health management in hazardous industries. The key challenges and recommendations associated with such use were also explored.

**Method:**

Using the Preferred Reporting Items for Systematic Reviews and Meta-Analyses (PRISMA) protocol, a total of 48 studies were reviewed to provide a framework for future pathways for the effective implementation of these innovations.

**Findings:**

The results revealed four main categories of digital safety systems: wearable-based systems, augmented/virtual reality-based systems, artificial intelligence-based systems, and navigation-based systems. A wide range of technological, behavioral, and organizational challenges were identified in relation to the key themes.

**Conclusion:**

Outcomes from this review can inform policymakers and industrial decision-makers about the application of digital innovations for best safety practices in various hazardous work conditions.

## Introduction

Digital innovations include real-time communication, big data, Internet of Things (IoT), man-machine cooperation, remote sensing and control, autonomous equipment, robots, artificial intelligence (AI), augmented reality (AR), virtual reality (VR), and production systems (Adriaensen et al., 2019; Badri et al., 2018; Pauliková et al., 2021). These technologies have contributed significantly to hazard identification, risk monitoring and controlling, and the overall safety of workers at high-risk workplaces (Adjiski et al., 2019; Greenfield et al., 2016; Rey-Merchán et al., 2021; Thibaud et al., 2018; Yang et al., 2020).

Previous studies have documented the utilization of innovative digital technologies for purposes such as equipment health monitoring, fault diagnosis, fleet monitoring, and preventive maintenance (Colantoni et al., 2018; Nnaji & Awolusi, 2021). In addition, the use of IoT with advanced sensor networks and control systems has served as leading indicators that provide real-time feedback for safety hazard identification and proactive response (Costin et al., 2019; Pishgar et al., 2021; Zhou et al., 2017). Research considers such digital advances as a solution to the current limitations associated with the traditional methods of managing occupational safety and risk management (Mouras & Badri, 2020; Nnaji & Awolusi, 2021). Previous reviews (e.g., Awolusi et al., 2018; Li et al., 2018; Pishgar et al., 2021; Thibaud et al., 2018) have explored the application of IoT and various technologies in enhancing decision-making processes within high-risk environments, particularly in health and safety industries such as health care, the food supply chain, mining, energy (including oil & gas and nuclear), intelligent transportation (e.g., connected vehicles), and building and infrastructure management for emergency response operations. However, these reviews were often limited to specific types of technologies within particular industrial contexts.

Moreover, there is still little known about their usefulness in promoting the safety of workers (Dodoo & Al-Samarraie, 2021; Dodoo et al., 2023; Laciok et al., 2021). Adriaensen et al. (2019) argued that the introduction of advanced technological innovations has introduced another layer of complexity into the existing socio-technical system and maintained that there is no one-solution-fits-all method for safety management in the workplace. This provides a firm research ground for further scrutiny of the usefulness of digital technologies for promoting workers’ safety and health in hazardous work environments. This includes the gap in understanding the current technological issues for safety management in high-risk industries. Therefore, the objective of this study was to develop a framework to categorize the range of digital technologies for safety management in hazardous environments and to understand their relevance for managing the safety and health of workers. Two main research questions were explored in this review: (1) how are digital innovations for safety and health used in hazardous industries? and (2) what are the key challenges and recommendations associated with such use?

## Method

The screening and review process of previous articles was carried out in accordance with the PRISMA (Preferred Reporting Items for Systematic Review and Meta-Analyses) 2020 statement (Page et al., 2021). In the context of this study, digital innovations/technologies were defined as the use or utilization of real-time communication, big data, IoT, man-machine systems, remote sensing and control, autonomous equipment, AI-based systems, AR and VR systems for managing safety and health of workers in hazardous industries. The classification of hazardous industries in this study was based on workplaces with a high probability of generating risks.

### Eligibility Criteria

The inclusion criteria adopted in this study consisted of (1) a publication date between 2017 and 2022, (2) written in English, (3) being an empirical study, (4) investigating the use of digital innovation solutions (e.g., AR, VR, AI, and IoT), and (5) the scope was in hazardous occupations (e.g., construction, oil and gas, manufacturing, and mining.

### Database Search

Science Direct, Web of Science, and ProQuest databases were used as the main source for searching and retrieving the relevant full-text English-language articles on the use/utilization of different digital innovations for health and safety management using the following phrases: (“online” OR “augmented reality” OR “virtual reality” OR “digital” OR “mobile” OR “electronic”) AND (“safety” OR “risk” OR “hazard*” OR “health management” OR “care” OR “protection” OR “aid”) AND (“workers” OR “employees” OR “laborers”) AND (“health management” OR “care” OR “protection” OR “aid”) published up to 2022. A complete update of the searches was accomplished in April 2022.

**Figure 1.**
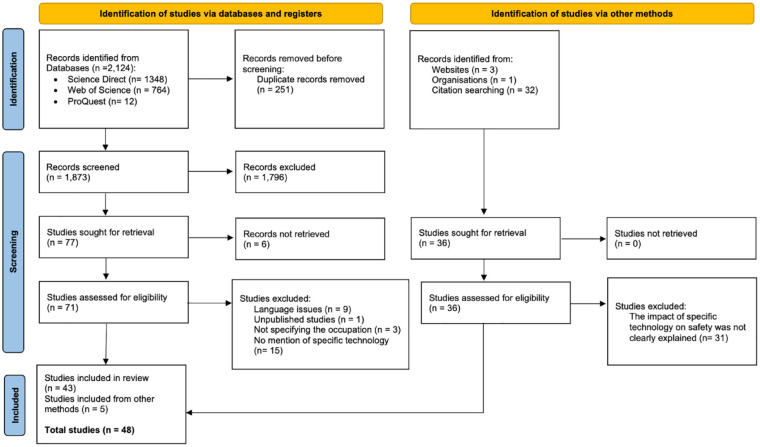
Article Searching and Selection Process Based on the PRISMA Statement.

In addition, we manually reviewed references of the retrieved articles to identify supplementary studies of interest. The search was restricted to full-text articles published in journal, conferences, proceedings, and book chapters. The subject of this search was limited to the relevant disciplines of industries, health and safety, technology at work, and technology engineering. The initial search resulted in 1,348 articles from Science Direct, 764 articles from Web of Science, and 12 articles from ProQuest. An additional 36 articles were found through reference searches, websites, and organizations. In total, this study examined 2,160 articles (see Figure 1).

### Study Selection

According to Figure 1, a total of 251 studies were excluded due to duplication, resulting in 1,873 articles remaining. After examining the titles and abstracts of these articles, we excluded 1,796 due to reasons such as irrelevance, being opinion-based, lacking quantitative/qualitative data, missing method information, lack of intervention, or no mention of technology. Consequently, only 77 articles were selected for retrieval, out of which 6 were unretrievable, leaving us with a total of 71 articles. These 71 articles were further processed using the developed eligibility criteria. We excluded studies based on language issues (e.g., the abstract was written in English, but the rest of the article was in another language) (*n* = 9), unpublished status (*n* = 1), failure to specify occupation (*n* = 3), and unclear explanation or absence of statistical evidence regarding the use of the adapted tool in safety management (*n* = 15). Through this process, we confirmed 43 articles for inclusion in this review. In addition, we included further articles using other methods (*n* = 36) and subjected them to eligibility checks. Out of the 36 studies, only 5 articles met the criteria, while others were excluded due to insufficient information on the direct impact of certain technologies on safety. Following these procedures, a total of 48 eligible empirical studies were included in this review (43 new studies included, and 5 studies identified through other methods), as shown in Figure 1. It is worth noting that our search did not identify articles specifically addressing the application of technologies to improve worker safety in domains commonly classified as hazardous, such as agriculture. This can be attributed to the primary focus of our study, which is to examine how technology informs safety practices in a broader sense, rather than focusing solely on worker safety within specific domains of application.

### Grouping the Findings of the Selected Articles

A literature matrix of multiple columns (study features) and rows (studies) was developed in this review to map the characteristics of the 48 eligible studies. The final list of articles was carefully read and independently evaluated by the authors based on the following criteria: (1) study purpose and country, (2) industry(s), (3) methodological characteristics (study design, type, and sample size), (4) technology characteristics (type of technology and purpose of use), (5) findings, and (6) challenges. We used a color-coding technique to compare codes along with an iterative process of discussion—which we used to agree on the naming and content of the codes (sub-factors). Through this coding process, four specific categories of systems were identified: wearable-based systems, augmented/virtual-based systems, artificial intelligence-based systems, and navigational-based systems. The identification of these categories was based on the type of technologies found in the collected articles, as well as their application to specific industrial contexts. Intercoder reliability was calculated to indicate the intercoder agreement for article classification across the authors. The obtained Krippendorff’s alpha for the four themes was above the recommended level of α = 0.8.

## Results

**Figure 2.**
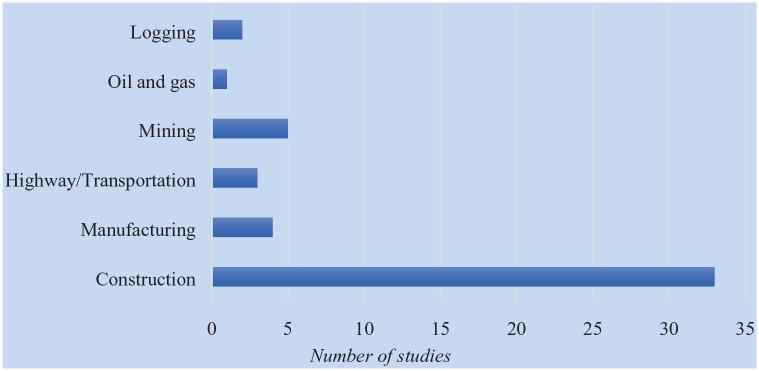
Distribution of Studies of Digital Innovations by Hazardous Industries.

The final list of empirical studies (*n* = 48 articles) included construction, mining, highway/transportation, manufacturing, logging, and oil and gas industries. Figure 2 shows the frequency of studies by industry sector. The majority of studies focused on the use of digital innovations for the safety and health of workers in the construction industry.

The retrieved papers were categorized according to the type of system used in the management of workers’ safety and health in high-risk occupations (Dodoo & Al-Samarraie, 2019). Further, the potential challenges and recommendations in relation to the use of digital innovations were identified. We grouped the findings into four key categories of systems, namely, wearable systems, AR/VR-based systems, AI-based systems, and navigation-based systems. These are explained in the following sections (see Table I in supplementary file for more details).

### Wearable-Based Systems

The study found that specific wearable systems were commonly used in the construction, transport, and mining industries. For example, in the construction industry, wireless sensors involving photo resistors, optical sensors, force stretchable resistors, and touch sensors were used to support the monitoring and controlling of proper use of Personal Protective Equipment (PPE) among workers. This innovative intervention averts potential violations and misuse of PPE among workers and reduces workplace accidents and injuries by sending warning signals to both user and safety officer through the IoT with wireless Wi-Fi modules tagged to the PPE.

In addition, battery-free sensing and communication prototypes, wearable computing, wearable inertial measurement, wrist wearable trackers, wearable sensors, smart vests, bands, rings, glasses, bracelets, safety boots, smartphones and smartwatches, electrodermal wrist sensors, exoskeletons/exosuits, WIPS-based gait parameters were also widely used in managing workers safety and health. These innovations have proactively prevented workplace accidents and enhanced the safety and health of workers in the construction industry. For instance, wearable sensors were useful for monitoring workers’ physiological health and safety behavior, improving awareness of hazards, and tracking their exposure to risky and hazardous substances in the work environment. In addition, a proposed virtual fence based on Bluetooth low-energy beacons was found to potentially improve construction safety by detecting and warning workers when they move into risk zones, as well as recording the time of exposure. This IoT-based system can potentially reduce fatalities associated with the industry due to fall from heights.

The review further indicated that mHealth wearable systems were used mainly in the highway/transportation industry to monitor the safety and health state of workers. For example, data from wearable devices were used to provide information for positive lifestyle modifications of drivers in the transportation industry, which helped reduce morbidity. For the mining industry, the results showed that IoT platforms such as Bluetooth-enabled heart rate monitors, smartphones, and smartwatches (energy-efficient Bluetooth sensors) were effective in monitoring workers’ exposure to harmful elements and proximity to danger zones, especially in underground mining. These IoT-enabled wearable systems have contributed to the management of workers’ safety and health.

### AR/VR-Based Systems

The results indicate that AR/VR-based systems were largely used in the construction and manufacturing industries. For example, the use of building information modeling (BIM) in construction projects was found to influence the promotion of safety and health of workers through design and proactive interventions. This includes identifying and eliminating potential hazards prior to the actual project implementation.

In addition, technologies such as AR systems (360-degree panoramas of reality) and VR systems were commonly used in the construction industry through the use of 3D and visual studio joint platforms interfaced with oculus rift. These innovations provided specific demonstration of safety practices by supporting the identification of potential hazards and risks prior to the actual implementation of a project. The review identified that for the manufacturing industry, a smart object digital prototype platform could be used to facilitate the tracking process of workers by creating a comprehensive picture of the whole process to protect them from plant hazards.

### AI-Based System

Our review showed that AI for workplace safety had been utilized mainly in industries like manufacturing, construction, mining, and oil and gas. The review identified a range of AI-based systems such as robotics and cobots which have been used for managing safety and health in the manufacturing industry. The literature reported that the uptake of robots of some aspect of the production process reduces the chances of humans having to take up harmful and unpleasant tasks, which can lead to improved workplace safety and quality of life. In addition, the construction industry used AI-based systems such as automatic data collection system, laser scanning and LiDAR, digital signage and robot and automation, cyber-physical systems, smart construction object, and digital lifting scaffold safety monitoring devices to minimize occupational hazards and risks.

In the mining industry, intelligent sensor and security technology were mainly used to offer efficient safety management through real-time tracking of worker’s locations and protection of underground miners. In the oil and gas industry, the gas lift oil network (soft sensors) proved to be useful in monitoring and controlling the production process. The system was found efficient for increasing the safety of the production process and improving the safety maintenance regime. Moreover, the review found that 3D LiDAR, 2D image data system, and real-time AI and AR interface design, and real-time wireless communication to be useful in reducing occupational hazards in the highway/transportation industry. These innovations have supported the effective tracking of potential risks and proactive response against collision, leading to increased safety of highway workers and vehicles.

### Navigation-Based Systems

We identified a range of digital innovative technologies utilized mainly in the construction, mining, logging, and highway/transportation industries. Systems used in the construction industry for controlling occupational hazards and risks range from real-time location-sharing technology, vision-based monitoring and image capture devices, unmanned aerial vehicles and photogrammetry, and radio frequency identification devices. These innovations support the early identification and elimination of potential hazards and risks, improved task performance, and technical support. Innovative systems such as radio frequency identification devices, ultra-wideband and 3D dynamic monitoring converters, and proximity detection sensors were used to reduce workplace hazards. These innovations support tracking of the mining process.

The application of other real-time location-sharing technologies and global satellite navigation systems (with radio frequency transmission) was mainly found in the logging industry. 3D LiDAR, 2D image data, and real-time AI and AR interface design were found to support the real-time tracking of the operational sites, improve workers’ alertness to danger, and prevent unauthorized access to the site.

Table 1 maps the utilization of specific digital innovations across industries. From the table, it can be noted that the majority of previous work has mostly concentrated on the use of various digital innovations in the construction industry. Technologies like smart/digital vest, wearable technology, AR and VR were commonly used in construction-related practices. Logging and highway were the least industries to explore how digital advances can increase the safety of workers. This can be due to the various challenges associated with the operation of these industries.

**Table 1.** The Utilization of Digital Innovations Across Industries

Digital innovations/industries	Construction	Mining	Manufacturing	Oil/gas	Highway/transportation	Logging
mHealth wearable devices					x	
Smart/digital vest/hat	x			x		
Smart wearable technology	x	x	x			
Virtual fences	x					
Wireless sensor devices	x	x	x	x		x
AR	x					
VR	x					
Building information modeling	x					
BIM 4D modeling	x					
Cobots			x			
Cyber-physical system	x					

*Note.* AR = augmented reality; VR = virtual reality; BIM = building information modeling.

## Discussion

### Wearable-Based Systems

Notable challenges were mainly found with the use of wearable systems for managing safety in the workplace. In the construction industry, we found that challenges were mainly technical, behavioral, and organizational in nature (see Table 2).

**Table 2.** Challenges and Recommendations Related to the Use of Digital Innovations in Workers’ Safety

System	Challenges	Recommendations	References
Wearable Systems	1. The battery-less device decreased the alert range. 2. High I-GAS values negatively impact the results. 3. The devices do not align with working conditions. 4. Invasion of privacy and the potential use of technology to monitor idling time. 5. The data is unreliable after a 4-day period. 6. Fluctuations in the UV index reading were detected. 7. Resistance to change. 8. Workers’ resistance, non-disclosure of medical conditions, and limitations with IoT connectivity. 9. The system does not align with job characteristics and high outdoor temperature. 10. The scope of application was limited to a laboratory setting and healthy volunteers. 11. There is a limited range of hazard detection on construction sites at night. 12. Lack of data to discretize findings, and demographic and trade factors affect the robustness of findings. 13. Privacy concerns and the quality of real-time information. 14. Insufficient dataset, concurrent postures impair the model’s performance, mental discomfort, and distractions. 15. Complex applications and limitations in working conditions. 16. It is not an energy-saving solution, high-capacity and heavy battery, raises false alarms, and fails to detect motions due to low illumination. 17. High noise and false alarms, and workers’ low risk perception.	1. Adding multiple antennas. 2. Devices could use workers’ abnormal gait responses to reveal hazards. 3. A tailor-made digital health system for truck drivers. 4. Educate workers on the safety and health benefits of using the technology. 5. Include job characteristics in the design. 6. Use a smartphone-based IoT gateway instead of a fixed location gateway. 7. Provide proper guidelines and information to facilitate acceptance. 8. Connect heart monitors to an internet-enabled network and use deep-learning algorithms to identify trends and forecast safety. 9. Develop an objective, continuous, and non-intrusive method to monitor workers’ physiological responses. 10. Conduct a real-world construction site experiment to broaden the scope of risk factors and use AR/VR to examine performance. 11. Increase the capability of the metal detection range. 12. Develop guidance to assist in the integration of useful technologies into mainstream practice. 13. Provide a framework and strategies to improve WSDs (Wireless Sensor Devices). 14. Sensor durability is required, and training should be provided to relieve workers of mental stress. 15. Connect wearable devices with specialized analytics on multi-data repositories. 16. Integrate modules and sensors into the system at real sites, test IP and IK codes, and improve core sensors to make it robust and stable. 17. Gain top management support, wide acceptance, and agreement with existing systems.	Teizer (2015); K. Yang et al. (2017); Greenfield et al. (2016); Choi et al. (2017); Lee et al. (2017); Wu et al. (2019); Callejas Sandoval and Kwon (2019); Costin et al. (2019); Antwi-Afari et al. (2020); Rajendran et al. (2020); Nnaji et al. (2021); Nnaji and Awolusi (2021); Zhao et al. (2021); Forat et al. (2021); Yang et al. (2020); Rey-Merchán et al. (2021)
AR/VR-based Systems	1. There is insufficient timing with the Google Glass-based BIM, a challenge with AR training, and resistance to change. 2. Ethical and privacy concerns affected the system’s effectiveness. 3. The BIM is not suitable for all projects, is costly to train personnel, and faces resistance to change. 4. There is limited time to identify hazards, low image quality, a static vantage point and stitching parallax, and the scoring approach lacks validity. 5. There is a lack of trust in the 4D system’s capacity to support safety management. 6. The technology did not consider sources of hidden errors. 7. The indicators used in risk warning are limited to the safety status of a specific location. 8. There is simulation sickness and disorientation in the virtual environment. 9. The challenges include cost, cultural barriers, lack of training, government policies, security concerns, and resistance to change.	1. Integrate BIM and AR technologies to increase wider technology acceptance. 2. Implement a BLE tracking algorithm and advanced sensory equipment. 3. Enhance communication when using the BIM to improve safety in the workplace. 4. Develop engaging training platforms for hazard identification, improve the platform to enhance the user experience, and improve validity. 5. Promote the use of 4D modeling for safety and health management. 6. The software requires wider implementation to improve it. 7. Improve systems engineering for wider coverage of the jobsite. 8. Adjust the navigation velocity to avoid simulation sickness; use mid-level light and neutral colors to prevent disorientation. 9. More knowledge and understanding of the BIM is needed to facilitate wide acceptance and use.	Teizer et al. (2017); Alomari et al. (2017); Eiris et al. (2018); Swallow and Zulu (2019); Deng et al. (2019); Joshi et al. (2021); Manzoor et al. (2021); Nnaji and Karakhan (2020)
AI-based Systems	1. There is a lack of balance between the abilities and interaction of cobots and humans. 2. There is wave propagation attenuation and loss of data. 3. Modeling speed, timeliness of data collection, and identification of unsafe behavior were not integrated into the system. 4. More modifications and improvements are still required. 5. The architecture processes were viewed as complex, ambitious, and impractical. 6. Values show false negatives due to obstructions in traffic. 7. There is resistance to continuous use, high cost of implementation and maintenance, low demand for the technology, lack of trust in the systems, and a lack of technical support such as training. 8. It is difficult to obtain a reliable degradation model for the equipment in real applications. 9. There are difficulties with linking resources, management, and social services against the allowable risk of performance. 10. There is a loss of connection between end nodes and the router if the distance is further than 60 m; RTT latency is impacted by the battery level of the smart glass, weather temperature, and heavy objects.	1. Create separate workspaces for robots and humans. 2. Improving accuracy and promptness can enhance the effectiveness of the systems. 3. Establish a more comprehensive smart construction site. 4. Implement automation, intelligent equipment, and digitalization to ensure construction safety. 5. Shifting in ideology is required for AI. 6. Improve the methodology. 7. Practitioners and designers should seek strategies to alleviate fears, enhance acceptance, and promote the use of technology to manage safety on jobsites. 8. Validate the approach by implementing the new model in an experimental rig. 9. A full transition to digitization of occupational health and safety control is necessary. 10. Further investigation is needed to measure the actual impact of the obstacles.	Pauliková et al. (2021); Kim et al. (2016); Jiang et al. (2021); Zhang (2021); Niu et al. (2019); Aijazi et al. (2017); Nnaji and Karakhan (2020); Matias et al. (2020); Sabeti et al. (2021)
Navigation-based Systems	1. The accuracy of the alert varies as the positions of workers and equipment change. 2. Detected delay time settings, lack of synchronization of mechanical equipment, and vibration effects. 3. Loss of hazard identification data and limitations with hardware. 4. Distraction and the cost of the technology. 5. Varying lighting conditions affect the accuracy of the algorithm. 6. Workers suffered from either over-trust or distrust in the mobile PDS. 7. There is resistance to continuous use, high cost of implementation and maintenance, low demand for the technology, lack of trust in the systems, and a lack of technical support such as training.	1. Implement correction methods to improve alert accuracy and utilize real-time positioning technology. 2. The system could be applied in small-scale settings, alongside excavators and other equipment. 3. It requires a fully connected networked environment. 4. Address the perception of potential distraction and the cost of the technology. 5. Combine guardrail detection with BIM to improve the system. 6. Designers should consider workplace characteristics. 7. Practitioners and designers should seek strategies to alleviate fears, enhance acceptance, and promote the use of technology to manage safety on jobsites.	Zimbelman et al. (2017); Jo et al. (2017); Kim et al. (2017); Newman et al. (2018); Kolar et al. (2018); Swanson et al. (2019); Nnaji and Karakhan (2020)

*Note.* UV = ultraviolet; AR/VR = augmented reality/virtual reality; BLE = bluetooth low energy; BIM = building information modeling; RTT = round trip time; AI = artificial intelligence; PDS = public distribution system.

#### Technical

Technical challenges observed were related to the accuracy of detecting changes in an environment upon encountering hazards. As such, future designs should consider worker’s abnormal gait responses to reveal safety hazards, as well as improve sensor durability and its applicability to the job characteristics of the construction industry. Other challenges in relation to wearable activity trackers, sensor network systems, heart rate monitoring, wristband sensors, smart vests, IoT wireless sensors, and SmartHat systems were also reported in the literature. This includes short lifespan of data, and fluctuations in the ultraviolet index reading. Meanwhile, limitations with IoT connectivity were found to negatively influence the system’s capacity to monitor worker’s level of exertion and health risks. These are essential in providing the necessary interventions that should safeguard the health and safety of workers.

We found that the use of limited range of hazard detection systems, complex/shared systems, and Wi-Fi modules is not suitable for certain industries due to the heavy high-capacity battery, false alarms, and non-detection of motion as a result of low illumination and decreased alert range. To improve the performance of these systems, devices should be assessed based on job characteristics to avoid data variability and adopt smartphone-based IoT gateways instead of dependence on fixed gateways. In addition, the process of enhancing the efficiency of monitoring workers’ stress in real time requires a greater range of individual monitoring mechanisms. It is believed that deep-learning algorithms can be useful in identifying trends and predicting workers stress levels for proper interventions. The review also suggests increasing the range of hazardous detection when using wearable technologies through the improvement of sensor durability, connecting devices with special analytic systems, and integrating other types of modules and sensors to provide robust and stable protection for workers’ health.

#### Behavioral

Behavioral challenges consist of workers’ concerns over invasion of privacy and fear that the wearable systems could be used to hunt down idling workers. In addition, resistance to change, lack of trust in technology, and non-disclosure of previous medical conditions are potential causes that prevent access to accurate data. These concerns can negatively impact workers’ adoption and continuous use of safety systems. To address these concerns, workers should be educated on the safety and health benefits of using safety systems. In addition, proper guidelines and strategic training programs on wearable and sensor-based technologies could relieve workers of the mental stress experienced due to the increased use of sensors. Such awareness could alleviate workers’ fears, create buy-in and facilitate technology acceptance.

#### Organizational

Organizational challenges that were identified with wearable systems include perceived lack of alignment of devices with job characteristics, thus not applicable to highly physically demanding activities and high-temperature outdoor environments. In addition, the lack of internet connectivity on jobsites, the limited scope of application of system, the lack of available demographic data and trade factors, and the cost of using and maintaining systems were also found to influence industries’ intention to adapt wearable systems in the safety management of workers. Therefore, customize wearable technologies that suits specific the characteristics of a specific industry could improve acceptance and usage. Meanwhile, a fully connected IoT on jobsites can improve the performance of wireless sensor devices. It is also believed that developing objective continuous and non-intrusive methods for monitoring workers’ physiological responses can help expand the scope of wearable technologies in real-world job environments.

### AR/VR-Based Systems

#### Technological

The review identified that BIM technology has been implemented mainly in the construction industry to support safety management. However, notable drawbacks were reported in terms of insufficient timing with the Google glass and AR training, the lack of capacity to track tasks being performed at workstations, and unsuitable for most construction projects. Another drawback of the BIM technology was its lack in evaluating hidden errors and risk warning indicators, which were limited to safety status of specific locations only. In this situation, training was important to improve the performance of safety systems. Our review showed that the current digital platforms require further evidence-based exploratory studies and wider implementation to assess their effectiveness in supporting construction site safety. We also identified drawbacks with the AR and VR training systems such as limited time to identify hazards, low quality of images, static vantage point and stitching parallax, lack of validity with the scoring approach, oculomotor disturbance, and disorientation. Such platforms require further enhancement and adjustment of navigation velocity to avoid oculomotor disturbance of user experience.

#### Behavioral

Workers’ resistance to change, cultural barriers, lack of skills and training were commonly addressed in previous studies. The review found that further engagement with workers and training should enhance workers’ technology acceptance. From an organizational perspective, the review found that ethical and privacy concerns hinder the use of AR/VR-based systems in safety management. In addition, the utilization of AR and VR technologies was perceived to be costly and requires direct capacity to support safety and health management, especially at construction sites. Again, the review showed that government policies, security concerns were major drawbacks toward the use of AR and VR systems on jobsites. It is recommended that future training programs that address the need of anti-technological workers could promote a wider usage of these technologies. In addition, further engagement with stakeholders on the integration of BIM with other technologies is necessary to promote technology acceptance.

### AI-Based Systems

#### Technological

The challenges with AI-based systems were mainly related to balancing the abilities and interaction of cobots and humans in a workspace context. Although the introduction of robots in the production process to handle harmful, monotonous, and high-risk tasks is necessary for improving the safety and quality of workers, the risk of potential injury to humans and the lack of coordination in the same workspace remains high. A consideration of a separate workspace for humans and robots to prevent accidents can provide an adequate remedy. Other drawbacks with AI applications were related to the lack of integrating wave propagation attenuation and loss of data, modeling speed, data collection timeliness and identification of unsafe behavior into the current systems.

To address these challenges, data accuracy and promptness are required to improve hazard identification and safety of workers. Further, difficulty with obtaining a reliable degradation model for equipment in real application was found to influence workers’ safety in the gas and oil well network. Moreover, implementing a new model in an experimental rig is essential.

#### Behavioral

Behavioral drawbacks of using AI-based systems include the architecture of the operation processes, which is largely perceived as complicated, unambitious, and impractical for controlling workers’ safety on construction sites. To address this, an ideological shift toward the place of AI in managing occupational safety and health is suggested. In terms of organizational drawbacks, the review revealed some difficulties in aligning resources and managing social services with future risks and performance. However, increasing safety levels require a full transition to digital OSH, especially in the mining industry, because it has a difficult work setting in order for workers to achieve maximum work results.

### Navigation-Based Systems

#### Technological

The study found a number of technical challenges in relation to the use of navigation-based systems in safety. For example, the accuracy of navigational systems (e.g., global navigation satellite system) varied when a worker or equipment position shifted to different angles. These variations in accuracy alerts may interfere with safety decision-making negatively. To improve the effectiveness of the system’s capacity to provide situational safety awareness to workers, manufacturers/developers must understand the factors that could lead to such variations and develop real-time positioning technology for accurate alerts of hazards in a workplace. Further, delay time settings and synchronization of mechanical equipment and vibration effect were reported as drawbacks when using proximity warning systems.

To address this challenge, the use of proximity warning systems could be limited to small construction fields. In addition, a fully connected networked environment is required to improve the capacity to monitor and warn workers of approaching hazards. Another technical challenge was related to varying lighting conditions on sites, which has the potential to affect the accuracy of future hazard detection models. This may impact negatively on the system’s capacity to monitor hazard locations and prevent unauthorized access to sites. To improve system’s effectiveness, a combination of guardrail detection with BIM is recommended.

#### Behavioral

Our review also revealed some behavioral challenges associated with the use of navigation-based systems in safety management. This includes either a lack of trust or distrust for proximity detection systems and resistance to change. These factors were found to slow down the smooth integration of navigation-based systems into specific job characteristics. To maximize the effectiveness of digital solutions against workplace hazards, organizations should consider enhancing operational integrity and the use of promotional materials to influence workers’ practices. The review identified other organizational challenges related to the cost of using and maintaining safety systems, lack of demand, doubt about the performance of technology and technical support. To sustain interest in the use of navigation-based systems for managing safety and health in the workplace, system developers must explore innovative ways to mitigate such uncertain situations.

## Study Limitations

This study was limited to previous research published between 2017 and 2022, potentially excluding older or more recent research that could contribute to a better understanding of the relationship between the use of digital innovation and workers’ safety. The eligibility criteria established in this study restrict the inclusion of only empirical research that specifically focuses on the utilization of real-time communication, big data, IoT, man-machine systems, remote sensing and control, autonomous equipment, AI-based systems, and AR and VR systems for managing the safety and health of workers in high-risk industries. Although the databases used in this study cover a large volume of research in the field, there is still a possibility of excluding relevant studies not indexed in those databases. Therefore, future studies are encouraged to conduct further research, taking into account and addressing the limitations identified in this study.

## Conclusion

This study sheds light on the current trend of utilizing digital technologies in managing occupational safety and health in high-risk occupations. The review found varied forms of using digital technologies in managing safety in the construction, mining, highway/ transportation, manufacturing, logging and oil and gas industries. For example, wearable-based systems were found to be effective in supporting safety behavior of workers (e.g., promoting proper use of PPEs, hazard awareness and identification), as well as monitoring and controlling workers’ physiological safety and health. Further, it has supported the tracking and controlling of work-related risks such as hazardous substances and workers’ exposure to them. The use of AR/VR-based systems was found to play a key role in offering proactive safety interventions through virtual prototype safety trainings. This study also addressed some technical, behavioral (e.g., invasion of privacy, workers’ low-risk perception and resistance to change), and organizational (e.g., lack of technology alignment and high cost of implementation) challenges. Therefore, industries are advised to consider investing in system upgrade and sustainable safety training programs to educate workers on the benefits of using the technology in their workplace. This can potentially result in an ideological shift toward technology acceptance among anti-technology workers. In addition, organizational-led effort toward smooth integration of technology into the industrial set-up could improve the use and safety efficiency of workers.

## Supplemental Material

sj-docx-1-whs-10.1177_21650799231215811 – Supplemental material for Digital Innovations for Occupational Safety: Empowering Workers in Hazardous Environments Supplemental material, sj-docx-1-whs-10.1177_21650799231215811 for Digital Innovations for Occupational Safety: Empowering Workers in Hazardous Environments by Joana Eva Dodoo, Hosam Al-Samarraie, Ahmed Ibrahim Alzahrani, Maria Lonsdale and Nasser Alalwan in Workplace Health & Safety
