# Supplementary material for: Digital Innovations for Occupational Safety: Empowering Workers in Hazardous Environments
Source: Workplace Health Saf. 2024 Jan 9;72(3):84–95. doi: 10.1177/21650799231215811 (PMC10928957; doi:10.1177/21650799231215811)
Supplement: sj-docx-1-whs-10.1177_21650799231215811 – Supplemental material for Digital Innovations for Occupational Safety: Empowering Workers in Hazardous Environments [file sj-docx-1-whs-10.1177_21650799231215811.docx]

**SUPPLEMENTARY FILE**

**TABLE I: List of Selected Studies**

| Wearable Systems | No | Study | Industry | Purpose | Digital solution/function | Findings |
| --- | --- | --- | --- | --- | --- | --- |
|  | 1 | Greenfield et al. (2017) | Highway/Transportation | Explored the views of professional truck drivers on mobile health technologies for promoting health and well-being | *System*: mHealth wearable devices  *Function:* monitoring vital parameters of workers | Workers expressed willingness to use mHealth technologies to prevent morbidity |
|  | 2 | Yang et al. (2017) | Construction | Proposed a collective sensing approach that senses and assesses workers’ gait abnormalities in order to identify physical fall hazards | *System*: A wearable inertia measurement unit attached to ankle to collect kinematic gait data  *Function*: tracking (monitoring and controlling of environmental parameters) | Results showed that the fall hazards such as obstacles and slippery surfaces generated disruptions in a worker’s gait patterns leading to falls |
|  | 3 | Choi et al. (2017) | Construction | Investigated the factors that determines construction workers’ adoption of wearable technologies in the workplace | System: Smart vest with an embedded indoor GPS, wrist wearable tracker with physiological sensors  Function: tracking and monitoring (location parameters of workers/ and monitoring vital parameters) safety and health support | Perceived usefulness, social influence and perceived privacy risk led to intention to adopt wearable technologies |
|  | 4 | Lee et al. (2017) | Construction | Assessed the reliability and usability of wearable sensors for monitoring roofing workers’ on-duty and off-duty activities | *Function*: Wearable sensors (activity tracker and physiological monitors)  *Function*: Tracking of activities and monitoring vital parameters | Wearable sensors were usable and provided reliable data for heart rate, energy expenditure, metabolic equivalents and sleep efficiency. |
|  | 5 | Jebelli et al. (2018) | Construction | Proposed the use of wearable sensors to detect workers’ physical and mental states during their work | *System*: Wearable sensors (off-the-shelf wristband-type)  *Function*: Monitoring vital parameters of workers | The use of the wristband-type wearable sensor helped at analyzing workers’ physical and mental states leading to early detection of stressor factors on site |
|  | 6 | Wu et al. (2019) | Manufacturing | Proposed a wearable sensor network system for Internet of Things (IoT) application for monitoring industrial safety | *System*: Wearable sensor network system  *Function*: Monitoring of both vital and environmental parameters | The wearable sensors attached to the body helped at monitoring physiological and environmental elements and provided valuable data for safety monitoring |
|  | 7 | Callejas Sandoval and Kwon (2019) | Construction | Investigated how smart wearable technology to support workplace safety among aging construction | *System*: Smart wearable technology  *Function*: Monitoring of vital and environmental parameters of workers | Perceived risk, cost, trust and lack of knowledge impacted the acceptance of smart wearable technology |
|  | 8 | Adjiski et al. (2019) | Mining | Presented a prototype system which uses sensors attached to PPE to provide real-time safety situation awareness and predict health and safety incidents before they occur | *System*: Smartphones and smartwatches via energy-efficient Bluetooth sensors  *Function*: Monitor environmental parameters at the workplace (workers’ health, exposure to harmful elements, proximity to danger zones) | IoT enabled devices can optimize efficiency in all aspects of underground mining and increase workplace safety |
|  | 9 | Costin et al. (2019) | Mining | Presented a new method that uses the IoT to monitor workers exertion/stress in real time based on established thresholds | *System*: IoT-Active Leading Indicator system) Bluetooth-enabled heart rate monitor)  *Function*: Monitoring | The tool enabled the officers to schedule break regimes for workers |
|  | 10 | Antwi-Afari et al. (2020) | Construction | Compared the validity and reliability of measuring wearable insole pressure system (WIPS-based) gait parameters against wearable inertial measurement units-based gait parameters for distinguishing safety hazards | System: Insole pressure system (WIPS-based gait parameters)  *Function*: Supporting the physical capabilities of workers | WIPs was comparable to the WIMU. Also, WIPs was a relevant noninvasive wearable sensing system for identifying safety hazards and preventing no-fatal fall injuries on jobsites |
|  | 11 | Rajendran et al. (2020) | Construction | Integrated intelligent systems into the design of PPEs to increase the efficiency of existing PPEs | *System*: Smart safety vest (metal detectors)  *Function*: Monitoring and controlling of environmental parameters to prevent emergencies | The use of metal detectors induced beeping sound when there is metal hazard around |
|  | 12 | Nnaji et al. (2021) | Construction | Investigated workers experiences on the use of wearable sensing devices for monitoring safety and health as well as mitigating risks among workers | *System*: Wearable sensing devices (Smartwatch/ band/ring/bracelet, Smart Safety Vests/Glasses, Boots etc.  *Function*: Monitoring and controlling of vital and environmental parameters | Wearable devices were efficient in improving workers’ safety and health such as detecting workers proximity to hazards |
|  | 13 | Okpala et al. (2021) | Construction | Examined workers’ acceptance of wearable sensing devices | *System*: Wearable sensing devices  *Function*: Monitoring vital parameters | Workers’ experience with wearable devices was found to predict their behavioural intention and actual use of technology for safety |
|  | 14 | Nnaji and Awolusi (2021) | Construction | Investigated the success factors for implementing wearable sensing devices for safety and health monitoring | *System*: wearable sensing devices  *Function*: safety monitoring and control (vital parameters of workers) | Education and training of workers, promoting personalized WSD and conducting detailed and continuous assessments of WSD as key strategies to improve the implementation irrespective contingency |
|  | 15 | Zhao et al. (2021) | Construction | Investigated the design of an effective wearable sensing system for musculoskeletal disorder prevention among workers. | *System*: Wearable inertia measurement unit sensing system  *Function*: Supporting (increasing the physical capabilities of workers) | The prototype design showed that wearable sensing system was a great approach for improving workers’ safety awareness (collecting motion data such as low discomfort and posture risk) |
|  | 16 | Forat et al. (2021) | Construction | Investigated use of wearable devices to collect unexpected data on potential risk at job site | *System*: Wearable devices  *Function*: Monitoring of environmental parameters (location of workers and machinery) at workplace | Data from the wearable devices assist with mitigating risks and creating optimal safety management system. It enabled early discovery of risks on site and a reduction in project delays |
|  | 17 | Yang et al. (2020) | Construction | Developed and experimented an automated PPE-Tool pair checking system to warn the user and safety officer on non-use of PPE | *Systems*: Internet of things (IoT) wireless sensors (contains photoresistors, optical sensors, force stretchable resistors and touch sensors)  *Function*: tracking (monitoring and controlling of safety and to create a comprehensive picture of the whole process) | The tool offered an early intervention for high-risk work events |
|  | 18 | Teizer (2015) | Construction | Assessed the capability of self-monitoring alert and reporting technology for hazard avoidance and training (SmartHat) technology | *System:* SmartHat system (battery-free radio frequency) that senses proximity to potential hazards.  *Function*: tracking (monitoring and control and proximity detection) | SmartHat when deployed as a wearable technology in PPE was reliable and effective in notifying ground workers in various hazard proximity positions and orientations |
|  | 19 | Nnaji and Karakhan (2020) | Construction | Identified technologies for managing safety and health in a workplace | *System*: BIM, Mobile Devices Onsite, Radio Frequency Identification, Augmented Reality, Virtual Reality  *Function*: Supporting physical capability of workers, simplification of information management and performing industrial designs  (support) | The use of these tools can improve workers’ awareness of hazards, and eliminating potential hazards at the design phase |
|  | 20 | Rey-Merchán et al. (2021) | Construction | Designed and evaluated a new system of virtual fences to prevent occupational accidents | *System*: Virtual fences based on Bluetooth low-energy  *Function*: Tracking (monitor location parameters of workers | Results showed that the system is less expensive and easy to integrate and configure |
|  | 21 | Le et al. (2015) | Construction | Proposed a wearable computing-based BIM and Augmented Reality to enhance the effectiveness of real-time safety management on-site. | System: BIM and Augmented Reality (AR) based system  *Function*: automated safety planning, experiential safety training, and real time safety monitoring and control | The system can potentially enhance the construction safety and proactively prevent workplace accidents |
|  | 22 | Teizer et al. (2017) | Construction | Proposed an Internet-of-Things (IoT) approach that integrates environmental and localization data in a cloud-based BIM platform for managing safety | System: Prototype system (BLE beacons, sensor-enhanced personal protective equipment (PPE), Building information modeling (BIM) and IoT platform.  *Function*: supporting (performing industrial designs) | The system can collect and visualize actual project data in real-time |
| Augmented and Virtual Reality Systems | 23 | Alomari et al. (2017) | Construction | Investigated the factors that enhances safety professionals believe in BIM for increasing safety in the work place | System: Building information modelling  *Function*: Supporting (performing virtual industrial design) | The increased use of BIM in the construction has led to better safety performance |
|  | 24 | Eiris et al. (2018) | Construction | Developed and evaluated a platform using augmented 360-degree panoramas of reality for safety-training to enhance hazard identification skills of trainees | *System*: Augmented 360-degree panoramas of reality (PARS)  *Function*: Training | Both platform and augmentations have improved significantly learning hazard identification among trainees |
|  | 25 | Swallow and Zulu (2019) | Construction | Investigated perceptions of industry professionals regarding the benefits and barriers of the adoption of 4D modeling for management of safety | System: BIM 4D modeling  *Function*: Supporting (performing industrial design) | The use of BIM 4D modeling can be influenced by cost of training, time to implement, and other cultural issues) |
|  | 26 | Deng et al. (2019) | Construction | Created a safety management module to simulate the emergency rescue of construction safety accidents and formulate the corresponding emergency management plan | *System*: BIM technology based on Revit platform (Navisworks software)  *Function*: Tracking (monitors location parameters of workers and assist to efficiently organize emergency evacuation) | The security management module was highly operational, easy to use, and offer real-time data update |
|  | 27 | Lin et al. (2019) | Construction | Proposed an integrated framework for closed-loop management of safety based on multisource data integration for structural safety during construction | *System*: Bridge-safety information model (BrSIM), Algorithms for data integration (3D products, schedule, structural simulation and monitoring from engineering systems)  *Function:* Supporting (simplification of information management and structural safety analysis) and monitoring environmental parameters | The BrSIM was useful for continuous integration of the structural analysis results and monitored data for the entire closed-loop management of safety planning, execution, safety monitoring, risk warning and assessment |
|  | 28 | Gnoni et al. (2020) | Process/ Manufacturing | Described a prototype system where Smart Objects integrate different IoT technologies and interacts through a digital platform to manage different hazards of plant workers | *Systems*: Smart object  *Function*: Tracking (create a comprehensive picture of the whole process) | The prototype was useful and relevant for protecting workers from occupational safety and health hazards |
|  | 29 | Joshi et al. (2021) | Construction | Developed a virtual reality for cost effective safety training to reduce exposure of employees to harm | *System*: VR with Oculus Rift/Oculus S  *Function*: Training (VR module to train safety protocols) | The VR module was user-friendly and offered minimal simulation sickness |
|  | 30 | Manzoor et al. (2021) | Construction | Explored how BIM and digital technologies could be used to mitigate safety hazards in high-rise building projects | *System*: AR/VR  *Function*: Monitoring environmental parameters and tracking (creating a complete picture of the whole process) | Users were motivated to use photogrammetry via AR/VR in high-rise building projects to increase safety |
|  | 31 | Pauliková et al. (2021) | Manufacturing | Used the SWOT analysis to ascertain the relevant tools for robotization of production processes in relation to occupational safety and health. | *Systems*: Robotics and cobots  *Function*: Supporting | Robotization of the production process can reduce workers’ exposure to harmful or hazardous elements |
|  | 32 | Kim et al. (2016) | Construction | Developed a model to automatically detect hazards on the path of workers to enhance safety management at the jobsite | *Systems*: Automatic data collection model  *Function*: Detection | The model identified hazards automatically and assisted in decreasing the time workers are exposed to them |
| AI Systems | 33 | Jiang et al. (2020) | Construction | Proposed a smart construction site framework for safety management | *System*: Cyber-physical system  *Function*: supporting risk data synchronizing mapping | The system was useful at creating risk data synchronization between the virtual construction and physical construction sites |
|  | 34 | Matias et al. (2020) | Oil and gas | Proposes a model predictive control approach for process monitoring and predictive maintenance paradigm | *System:* gas lift oil well network (via a soft sensor)  *Function:* for monitoring and control | Production increased while making sure that the critical levels of erosion are not exceeded (safe operations) |
|  | 35 | Zhang (2021) | Construction | Developed a digital safety monitoring device for lifting scaffold and safety monitoring control device. | *System*: Digital lifting scaffold safety monitoring device  *Function*: Supporting (monitoring and controlling, provides a risk pre-warning caused by load) | The model offered simple structure, convenient operation, and a strong guarantee for the construction safety of the attached lifting scaffold |
|  | 36 | Niu et al. (2019) | Construction | Developed a smart construction object occupational health and safety system to enhance safety performance | *System*: Artificial intelligence  *Function*: Monitoring and tracking of environmental parameters and taking action to avoid hazards | The system identified dangerous situations and responded to them autonomously |
|  | 37 | Savon, Aleksakhin, Skryabin, and Goodilin (2019) | Mining | Discussed digitization as a solution to occupational health and safety related issues in the coal industry | *System*: United Automated Control Center information and analysis system  *Function*: Support and control | The digitization has reduced the lost time injury frequency and increased the safety performance |
|  | 38 | Xie et al. (2021) | Mining | Adapted the heuristic mathematical model based on IoT and RFID real-time monitoring system for production and mine safety and analysis | *System*: RFID, sensor, intelligent and security technologies  *Function*: Tracking | The system allowed for real-time tracking, detecting suspicious incidents, and verification of the position of a miner within the harsh underground mining environment |
|  | 39 | Aijazi et al. (2017) | Highway/ Transportation | Proposed an automatic obstacle detection and tracking system for anti-collision management | *System*: 3D LiDAR and 2D image data  *Function*: Tracking | The system prevented struck by machinery, as well as detecting and avoiding collisions |
|  | 40 | Sabeti et al. (2021) | Highway/ Transportation | Presents an integrative design framework of AI and AR to enhance the safety of highway workers | *System:* real-time AI and AR interface design & real-time wireless communication  *Function*: Tracking | The AI model helped at detecting vehicles from distance with 24.83 Frame per seconds |
|  | 41 | Zimbelman et al. (2017) | Logging | Evaluated the factors affecting mobile geofence accuracy to determine the viability of virtual safety zones for improving situational awareness | Global navigation satellite system technology paired with radio frequency transmission (GNSS-RF) (used to reduce fatal and non-fatal accidents in logging operations.  *Function*: Tracking (monitor location parameters of workers and prevent unauthorized access to worksite) | The system monitored location parameters of workers and prevent unauthorized access to worksite |
|  | 42 | Jo et al. (2017) | Construction | Evaluated the applicability of a proposed proximity warning system to address issues of collision and enhance safety | *System*: Proximity Warning and Excavator Control System (with radio frequency identification sensor RFID)  *Function*: Tracking | The system was found useful at detecting approaching worker where the excavator instantly shuts down to avoid collision |
| Navigation-based Systems | 43 | Kim et al. (2017) | Construction | Proposed a vision-based hazard avoidance system to proactively inform workers of potentially dangerous situations | *System*: Vision-based monitoring module, image capture device that uses fuzzy-based reasoning  *Function*: Tracking (monitoring location parameters of workers) | The system could potentially mitigate hazards and improve safety at the workplace |
|  | 44 | Newman et al. (2018) | Logging | Investigated the human factors and how real-time location-sharing technology can be used to mitigate incidents and improve situational awareness for loggers. | *System*: Real-time location-sharing technology  *Function*: Monitoring and risk assessment; tracking incidents | The technology was found to improve safety by alerting workers of potential hand-faller injuries due to lack of movement |
|  | 45 | Kolar et al. (2018) | Construction | Assessed the potential benefits of information and sensing technologies for onsite safety inspection | *System*: Safety guardrail detection model based on convolutional neural network  *Function*: Tracking | The synthetic images generated by the augment technology can be useful for monitoring location parameters and prevent unauthorized access to sites |
|  | 46 | Sun et al. (2018) | Mining | Proposed a safety risk control system based on UWB and 3D visualization in metallurgical operation. | *System:* Ultra-wide Band and 3D dynamic monitoring converter  *Function*: Tracking (monitoring of location parameters of workers and to prevent unauthorized access to operational sites) | The system provided comprehensive control and monitoring services. It improved safety of metallurgical personnel, reliability of security check and man-machine orderly cooperation at work |
|  | 47 | Swanson et al. (2019) | Mining | Examined how the type of training offered influences mineworkers trust in automated systems for managing occupational safety and health | *System*: Proximity detection systems (PDS) for mobile machines  *Function*: Tracking (preventing struck by machinery and object proximity detection) | The characteristics of the operating mine were significantly related to workers’ trust in mobile PDSs |
|  | 48 | Nnaji and Karakhan (2020) | Construction | Identified technologies for managing safety and health as well as the benefits and barriers to their application in the construction industry | Mobile Devices Onsite, Unmanned Aerial Vehicles, Photogrammetry, Radio Frequency Identification  *Function*: Supporting physical capability of workers, simplification of information management and performing industrial designs  (support) | The results revealed a marginal increase in these technologies for improving workers’ awareness of hazards, and eliminating potential hazards in the workplace. |
